# Gastric cancer nodal tumour–stroma ratios influence prognosis

**DOI:** 10.1002/bjs.12054

**Published:** 2020-10-14

**Authors:** J. Huang, B. Yang, J. Tan, S. Zhou, Z. Chen, G. Zhong, H. Gao, J. Zhu, J. Zeng, L. Zhong, X. Liu, F. Han

**Affiliations:** ^1^ Department of Gastrointestinal Surgery Sun Yat‐sen Memorial Hospital, Sun Yat‐sen University Guangzhou China; ^2^ Zhu Jiang Hospital of Southern Medical University, Southern Medical University Guangzhou China

## Abstract

This study showed that nodal tumour–stroma ratio (NTSR) is an independent prognostic factor for overall and disease‐free survival of patients with gastric cancer. Both relative stroma‐rich primary tumour–stroma ratio (PTSR) and NTSR were independent negative prognostic factors for overall survival in gastric cancer. This study supports assessment of tumour–stroma ratio in the routine pathological diagnosis of gastric cancer.

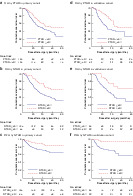

validated in node‐positive disease

## Introduction

Gastric cancer is one of the most common digestive tumours^1^. An ideal pathological staging system should not only reflect the biological characteristics of the tumour, but also be reproducible and clinically applicable. In 2007, Mesker and colleagues^2^, ^3^ proposed the concept of tumour–stroma ratio (TSR) as the proportion of tumour cells relative to surrounding interstitial components. TSR is the most macroscopic index used to evaluate the tumour microenvironment. Primary TSR (PTSR) may be an independent prognostic factor that predicts the prognosis of various solid tumours such as hepatocellular, breast, upper and lower gastrointestinal cancers^4^, ^5^, ^6^, ^7^, ^8^, ^9^, ^10^, ^11^, ^12^, ^13^, ^14^, ^15^, ^16^. Few studies have examined the prognostic value of nodal TSR (NTSR) in depth, so this analysis explored its validity in gastric cancer.

## Methods

A complete description of the study design, TSR evaluation and statistical analysis is available in *Appendix* *S1* (supporting information). In brief, this retrospective study evaluated the clinical significance and prognostic value of NTSR in gastric cancer. All procedures performed were in accordance with the ethical standards of the responsible committee on human experimentation (institutional and national), and with the Helsinki Declaration of 1964 and later versions. Informed consent was obtained from all patients.

## Results

A total of 708 consecutive patients with gastric adenocarcinoma and metastatic lymph nodes after radical gastrectomy were included in our study. Between January 2011 and December 2015, 468 patients were recruited at Sun Yat‐sen Memorial Hospital for the internal training cohort and 240 patients were recruited at Zhu Jiang Hospital of Southern Medical University for the external validation cohort. Following exclusions (*Fig. S1*, supporting information), 260 patients in the primary cohort and 129 in the external validation cohort were included in the study (*Table S1*, supporting information).

### Nodal tumour–stroma ratio score and optimal cut‐off value

The NTSR score was determined following the principles of PTSR assessment^17^ (*Fig. S2*, supporting information). Assessment of NTSR was done as follows: when micrometastases were present, the proportion of stroma was evaluated in a smaller image field as long as tumour cells were present at all borders; lymph node organs such as lymphoid follicles were not considered as stromal components; blood vessels regardless of size were included in the interstitial components; and in patients with multiple lymph node metastases, the NTSR assessment was first done on the largest metastatic lesion that could be evaluated for TSR using a 40‐fold objective.

**Fig. 1:**
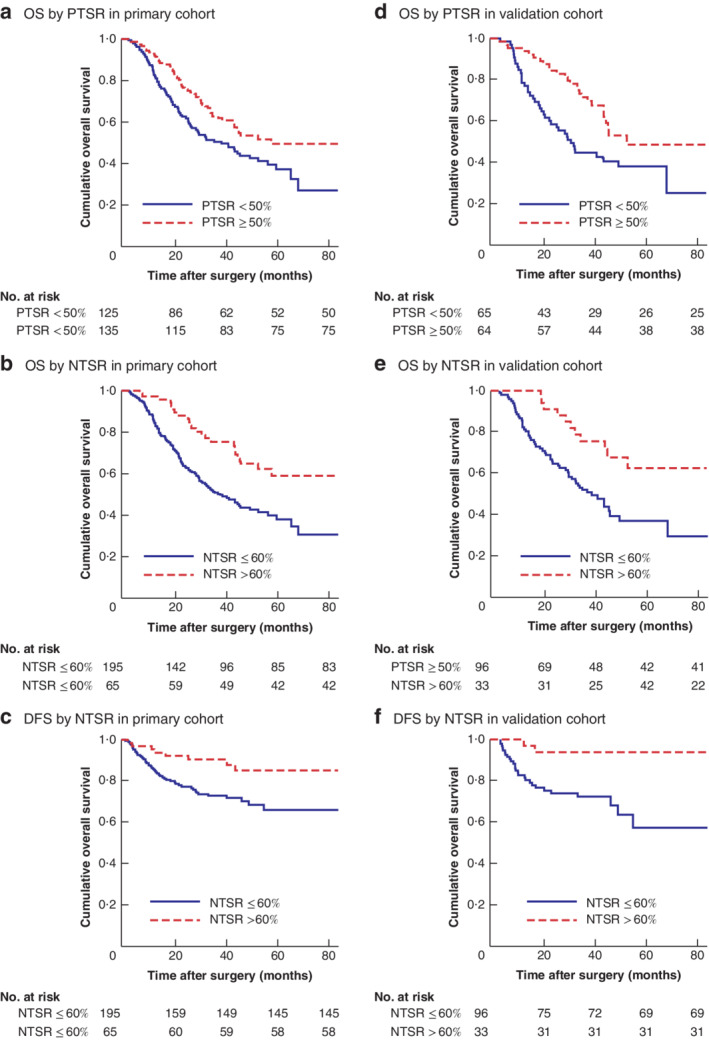
Kaplan–Meier survival curves according to primary and lymph node tumour–stroma ratio in primary and external validation cohorts **a** Overall survival (OS) according primary tumour–stroma ratio (PTSR), **b** OS according nodal tumour–stroma ratio (NTSR) and **c** disease‐free survival (DFS) according to NTSR in primary cohort; **d** OS according to PTSR, **e** OS according to NTSR and **f** DFS according to NTSR in validation cohort. **a** *P* = 0·013, **b** *P* = 0·001, **c** *P* = 0·009, **d** *P* = 0·007, **e** *P* = 0·005, **f** *P* = 0·004 (log‐rank test).

**Fig. 2:**
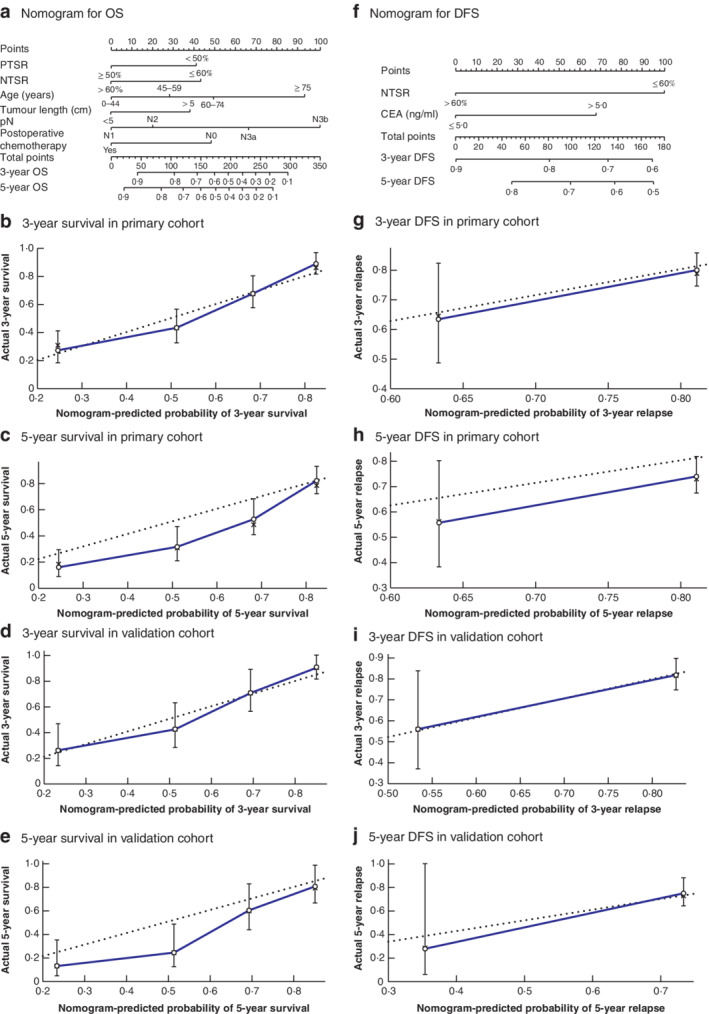
Evaluation of integrated systemic nomograms for overall and disease‐free survival **a** Nomogram and **b–e** calibration curves for 3‐year (**b**,**d**) and 5‐year (**c**,**e**) overall survival (OS) in the primary (**b**,**c**) and external validation (**d**,**e**) cohorts. **f** Nomogram and **g–j** calibration curves for 3‐year (**g**,**i**) and 5‐year (**h**,**j**) disease‐free survival (DFS) in the primary (**g**,**h**) and external validation (**i**,**j**) cohorts. In the nomogram, the value for an individual patient is located on the axis for each variable, and a line is drawn upwards to determine the points received for each variable. The sum of these scores is located on the total points axis, and a line is drawn downwards to the survival axis to determine the likelihood of 3‐ or 5‐year OS or DFS. Error bars are 95 per cent c.i., crosses are the Kaplan–Meier corrected mean.

A receiver operating characteristic (ROC) curve was used to analyse the relationship between NTSR and overall survival (OS). The area under the ROC curve was 0·581 (95 per cent c.i. 0·512 to 0·651; *P* = 0·023). Using the Youden index, the optimal cut‐off value for NTSR was found to be 0·65. As the NTSR index was scored in increments of 10 per cent, the optimal cut‐off value of NTSR was set as 0·60, with NTSR divided into over 60 per cent (stroma low) and 60 per cent or less (stroma high).

### Impact of primary and nodal tumour–stromal ratio on survival

Results of Kaplan–Meier survival analysis for the primary cohort are shown in *Fig*. *1* and *Table S2* (supporting information). PTSR below 50 per cent was a negative predictor of OS (*P* = 0·013) (*Fig*. *1a*). NTSR of 60 per cent or less was a negative predictor of OS (*P* = 0·001) and disease‐free survival (DFS) (*P* = 0·009) (*Fig*. *1b*,*c*). Cox univariable and multivariable analyses confirmed that PTSR and NTSR were independent prognostic predictors of OS (*Table* *1*). Because the patients in this study had gastric cancer and lymph node metastasis, the majority had a ypTNM stage of III. Stratified analysis showed that NTSR was a good predictor of OS (*P* = 0·002) and DFS (*P* = 0·019) in patients with ypTNM stage III disease. Cox multivariable analyses showed that, in addition to PTSR and NTSR, age, pN status, tumour length and postoperative chemotherapy were independent risk factors associated with OS in gastric cancer. The results of Cox univariable and multivariable analyses of DFS are shown in *Table S3* (supporting information). Only carcinoembryonic antigen level and NTSR were independent risk factors for DFS in gastric cancer.

**Table 1:** Results of Cox univariable and multivariable analyses for overall survival in primary cohort

	Univariable analysis		Multivariable analysis including PTSR		Multivariable analysis including NTSR		Multivariable analysis including PTSR and NTSR	
	Hazard ratio	*P*	Hazard ratio	*P*	Hazard ratio	*P*	Hazard ratio	*P*
**Age (years)**		0·059		0.008		0·066		0·021
<45	0·37 (0·17, 0·79)		0·30 (0·14, 0·66)		0·39 (0·18, 0·85)		0·32 (0·14, 0·71)	
45–60	0·56 (0·32, 0·97)		0·41 (0·22, 0·74)		0·53 (0·29, 0·95)		0·46 (0·25, 0·84)	
60–75	0·65 (0·38, 1·11)		0·54 (0·31, 0·94)		0·67 (0·39, 1·17)		0·59 (0·33, 1·04)	
> 75	1·00 (reference)		1·00 (reference)		1·00 (reference)		1·00 (reference)	
**Sex**		0·249						
M	1·23 (0·86, 1·76)							
F	1·00 (reference)							
**pN status**		< 0·001		< 0·001		< 0·001		< 0·001
pN1	0·24 (0·14, 0·41)		0·25 (0·14, 0·45)		0·28 (0·16, 0·52)		0·30 (0·16, 0·54)	
pN2	0·34 (0·21, 0·57)		0·34 (0·20, 0·58)		0·36 (0·21, 0·63)		0·37 (0·22, 0·65)	
pN3a	0·66 (0·41, 1·05)		0·62 (0·38, 1·00)		0·68 (0·42, 1·11)		0·66 (0·40, 1·06)	
pN3b	1·00 (reference)		1·00 (reference)		1·00 (reference)		1·00 (reference)	
**pT status**		0·009						
pT1/T2	0·39 (0·19, 0·79)							
pT3/T4	1·00 (reference)							
**ypTNM stage** *		0·011						
I	0·37 (0·12, 1·15)							
II	0·42 (0·21, 0·82)							
III	1·00 (reference)							
**Differentiation**		0·042						
Well/moderately	0·61 (0·37, 0·98)							
Poorly	1·00 (reference)							
**Tumour location** **†**		0·291						
Upper	1·00 (reference)							
Middle	0·82 (0·52, 1·31)							
Low	0·92 (0·61, 1·38)							
Total	1·90 (0·81, 4·49)							
**Tumour length (cm)**		<0·001		0·023		0·048		0·014
< 5	0·53 (0·38, 0·74)		1·00 (reference)		1·00 (reference)		1·00 (reference)	
≥ 5	1·00 (reference)		1·54 (1·06, 2·23)		1·46 (1·00, 2·11)		1·60 (1·10, 2·33)	
**CEA (ng/ml)**		0·301						
≤ 5·0	0·80 (0·52, 1·22)							
> 5·0	1·00 (reference)							
**Postoperative chemotherapy**		0·002		0·001		0·003		0·001
Yes	1·00 (reference)		0·56 (0·40, 0·80)		0·58 (0·41, 0·83)		0·56 (0·39, 0·80)	
No	1·72 (1·22, 2·43)		1·00 (reference)		1·00 (reference)		1·00 (reference)	
**PTSR (%)**		0·014		0·002				0·006
≥ 50	1·00 (reference)		1·00 (reference)				1·00 (reference)	
<50	1·53 (1·09, 2·15)		1·77 (1·24, 2·53)				1·65 (1·15, 2·37)	
**NTSR (%)**		0·001				0·008		0·026
> 60	1·00 (reference)				1·00 (reference)		1·00 (reference)	
≤ 60	2·16 (1·38, 3·39)				1·89 (1·18, 3·00)		1·71 (1·07, 2·74)	

Values in parentheses are 95 per cent confidence intervals.

* According to the eighth edition of the AJCC TNM system^18^.

† According to the Japanese classification of gastric carcinoma (3rd English edition)^19^. PTSR, primary tumour–stromal ratio; NTSR, nodal tumour–stromal ratio; CEA, carcinoembryonic antigen.

### Nomogram development and validation

Clinical characteristics, and the proportion of patients with low PTSR (below 50 per cent) and NTSR (60 per cent or less) values were similar in the primary and external validation cohorts (*Table S4*, supporting information).

A Cox proportional hazards regression model was constructed based on the Akaike information criterion, with backward stepwise selection, to find a best‐fit model. A nomogram comprising six independent factors was used to predict 3‐ and 5‐year OS of patients with gastric cancer (*Fig*. *2a*). The C‐index for the nomogram was 0·72 (95 per cent c.i. 0·70 to 0·74), higher than that for NTSR alone (C‐index 0·57, 0·56 to 0·59) and the ypTNM staging system (C‐index 0·64, 0·62 to 0·66). The nomogram showed good prediction performance for OS in patients with gastric cancer. Calibration plots for the probability of survival at 3 or 5 years after radical gastric cancer surgery showed good correlation between the value predicted by the nomogram and the actual observation (*Fig*. *2b*,*c*). The C‐index for the nomogram (*Fig*. *2f*) that predicted 3‐ and 5‐year DFS of patients with gastric cancer was 0·61 (0·58 to 0·64), and was better than that for NTSR alone (C‐index 0·58, 0·55 to 0·60) and the ypTNM staging system (C‐index 0·55, 0·53 to 0·57) indicated that the new model was effective in predicting DFS of patients with gastric cancer.

When the nomogram was subjected to external validation in an independent cohort, the C‐index was 0·75 (0·73 to 0·78) for OS and 0·66 (0·62 to 0·70) for DFS, which was greater than that for the current ypTNM staging system. The calibration plots also showed optimal agreements between nomogram predictions and actual observations for 3‐ and 5‐year OS and DFS in the external validation cohort (*Fig*. *2d*,*e*,*i*,*j*), confirming that the nomogram was an accurate and useful tool for the prediction of OS and DFS in patients with gastric cancer.

## Discussion

Gastric cancer cells are recirculated through lymphatic vessels and colonize in lymph nodes to form a unique lymph node metastatic microenvironment. Tumour‐associated stromal components may regulate tumour development, invasion and drug resistance by processes such as secreting protumour factors, inducing angiogenesis and promoting immunosuppression^20^.

The present study had some limitations including that it was retrospective, involved only node‐positive gastric cancer, and the nomograms included only basic clinical characteristics and pathological parameters. It did, however, confirm that relative stoma‐rich PTSR and NTSR indicated worse prognosis in patients with gastric cancer. Both PTSR and NTSR were identified as independent predictors of gastric cancer prognosis. The nomogram performed better than the ypTNM staging system. TSR is a simple, convenient and clinically significant pathological indicator that should be recommended as a routine pathological index.

## Supporting information


**Appendix** S1. Supoorting Information
