# Supplementary material for: Gastric cancer nodal tumour–stroma ratios influence prognosis
Source: Br J Surg. 2020 Oct 14;107(13):1713–8. doi: 10.1002/bjs.12054 (PMC7702113; doi:10.1002/bjs.12054)
Supplement: Supplementary file 1 — Appendix S1. Supoorting Information [file BJS-107-1713-s001.doc]

**BJS12054**

**Gastric cancer nodal tumour–stroma ratios influence prognosis**

J. Huang, B. Yang, J. Tan, S. Zhou, Z. Chen, G. Zhong, H. Gao, J. Zhu, J. Zeng, L. Zhong, X. Liu and F. Han

**Appendix S1 Materials and methods**

1. **Study population:**

The study retrospectively analyzed patients with gastric adenocarcinoma who underwent D2 radical gastric cancer surgery from January 2011 to December 2015 in Sun Yat-sen Memorial Hospital and Zhu Jiang Hospital of Southern Medical University. The exclusion criteria were patients who had distant metastasis or other history of malignant tumors, total number of lymph nodes removed less than 15, death within 30 days after surgery, and loss of follow-up. TSR assessment of primary gastric cancer and metastatic lymph nodes were performed for the remaining patients. This study approval was obtained from independent ethics committees from Sun Yat-sen Memorial Hospital and Zhu Jiang Hospital of Southern Medical University.

1. **TSR evaluation**

TSR was assessed by conventional light microscopy on 5 μm routine H&E stained slides. First, the deepest infiltration level in PT and LNs were evaluated with a 5x objective and the most stroma-rich tissue area(s) were identified. The most stroma-abundant area was selected and assessed with a 10x objective. Only tumor fields with tumor cells present at all borders of the image field were eligible. A primary lesion tumor-stroma ratio ≥50% was categorized as stroma-less and a TSR <50% was considered stroma-rich (Figure 1). The TSR of metastatic lymph node percentage was scored by increments of 10% (Figure 1). Lymph node metastases of >0.2 mm but ≤2 mm were defined as micrometastases. In case of micrometastases, the TSR was evaluated in a smaller image field as long as tumor cells were present at all borders.

1. **Statistical analysis**

IBM Statistics v24.0 (SPSS, Inc., an IBM Company Chicago, IL USA) and R software version 3.5.3 (http://www.r-project.org/) with the foreign and rms packages were used to perform statistical analyses. The chi-square test was used to compare the non-continuous baseline variable of the two groups, and the *t*-test for continuous variable. The survival analysis was performed by Kaplan-Meier (KM) and Cox univariate and multivariate proportional hazard analyses. The LNTSR cut-off value was selected by ROC curve analysis. The selection of cut-off point was the maximum of Youden index, which was defined as max_c_ [Sen(c) ＋ Spe(c) － 1]. For all analysis, *P* <0.05 was considered statistically significant. The nomogram curve was formulated based on the results of the multivariate Cox regression analysis by a backward stepdown selection process with the Akaike information criterion (AIC). The concordance index (C-index) and calibration curve were derived based on regression analysis. Additionally, the external validation cohort was used to validate the nomogram. All statistical tests were two-sided with a statistical significance level set at *p* value <0.05.

**Table S1** Patient characteristics and statistically significant difference between primary TSR and NTSR

| Items | Total N (%) | Primary TSR | | |  | NTSR | | |
| --- | --- | --- | --- | --- | --- | --- | --- | --- |
|  |  | ≥50% (n=135) | ＜50% (n=125) | *P*^a^ value |  | ＞60% (n=207) | ≤60% (n=53) | *P*^a^ value |
| Age |  |  |  | 0.251 |  |  |  | 0.313 |
| ＜45 | 32 (12.3%) | 15 (11.1%) | 17 (13.6%) |  |  | 7 (10.8%) | 25 (12.8%) |  |
| 45~60 | 86 (33.1%) | 40 (29.6%) | 46 (36.8%) |  |  | 25 (38.5%) | 61 (31.3%) |  |
| 60~75 | 117 (45.0%) | 63 (46.7%) | 54 (43.2%) |  |  | 30 (46.2%) | 87 (44.6%) |  |
| ＞75 | 25 (9.6%) | 17 (12.6%) | 8 (6.4%) |  |  | 3 (4.6%) | 22 (11.3%) |  |
| Gender |  |  |  | 0.102 |  |  |  | 0.711 |
| Male | 163 (62.7%) | 61 (67.4%) | 72 (57.6%) |  |  | 42 (64.6%) | 121 (62.1%) |  |
| Female | 97 (37.3%) | 44 (32.6%) | 53 (42.4%) |  |  | 23 (35.4%) | 74 (37.9%) |  |
| pN status |  |  |  | 0.112 |  |  |  | **0.012** |
| N1 | 83 (31.9%) | 50 (37.0%) | 33 (26.4%) |  |  | 30 (46.2%) | 53 (27.2%) |  |
| N2 | 77 (29.6%) | 42 (31.1%) | 35 (28.0%) |  |  | 18 (27.7%) | 59 (30.3%) |  |
| N3a | 68 (26.2%) | 28 (20.7%) | 40 (32.0%) |  |  | 14 (21.5%) | 54 (27.7%) |  |
| N3b | 32 (12.3%) | 15 (11.1%) | 17 (13.6%) |  |  | 3 (4.6%) | 29 (14.9%) |  |
| pT status |  |  |  | **0.003** |  |  |  | 0.914 |
| T1/T2 | 33 (12.7%) | 25 (18.5%) | 8 (6.4%) |  |  | 8 (12.3%) | 25 (12.8%) |  |
| T3/T4 | 227 (87.3%) | 110 (81.5%) | 117 (93.6%) |  |  | 57 (87.7%) | 170 (87.2%) |  |
| ypTNM stagingb |  |  |  |  |  |  |  | 0.472 |
| Ⅰ | 13 (5.0%) | 9 (6.7%) | 4 (3.2%) |  |  | 5 (7.7%) | 8 (4.1%) |  |
| Ⅱ | 33 (12.7%) | 25 (18.5%) | 8 (6.4%) |  |  | 9 (13.8%) | 24 (12.3%) |  |
| Ⅲ | 214 (82.3%) | 101 (74.8%) | 113 (90.4%) |  |  | 51 (78.5%) | 163 (83.6%) |  |
| Differentiation |  |  |  | 0.078 |  |  |  | 0.082 |
| Well/Moderately | 49 (18.8%) | 31 (23.0%) | 18 (14.4%) |  |  | 17 (26.2%) | 32 (16.4%) |  |
| Poor | 211 (81.2%) | 104 (77.0%) | 107 (85.6%) |  |  | 48 (73.8%) | 163 (83.6%) |  |
| Tumor locationc |  |  |  | 0.212 |  |  |  | 0.146 |
| Upper | 74 (28.5%) | 46 (34.1%) | 28 (22.4%) |  |  | 23 (35.4%) | 51 (26.2%) |  |
| Middle | 66 (25.4%) | 31 (23.0%) | 35 (28.0%) |  |  | 10 (15.4%) | 56 (28.7%) |  |
| Low | 113 (43.5%) | 55 (40.7%) | 58 (46.4%) |  |  | 30 (46.2%) | 83 (42.6%) |  |
| Total | 7 (2.7%) | 3 (2.2%) | 4 (3.2%) |  |  | 2 (3.1%) | 5 (2.6%) |  |
| Tumor length (cm) |  |  |  | 0.113 |  |  |  | 0.828 |
| ＜5 | 147 (56.5%) | 70 (51.9%) | 77 (61.6%) |  |  | 36 (55.4%) | 111 (56.9%) |  |
| ≥5 | 113 (43.5%) | 65 (48.1%) | 48 (38.4%) |  |  | 29 (44.6%) | 84 (43.1%) |  |
| CEA (ng/ml) |  |  |  | **0.008** |  |  |  | 0.691 |
| ≤5.0 | 215 (83.0%) | 104 (77.0%) | 111 (89.5%) |  |  | 55 (84.6%) | 160 (82.5%) |  |
| ＞5.0 | 44 (17.0%) | 31 (23.0%) | 13 (10.5%) |  |  | 10 (15.4%) | 34 (17.5%) |  |
| Postoperative chemotherapy |  |  |  | 0.130 |  |  |  | 0.943 |
| Yes | 135 (51.9%) | 64 (47.4%) | 71 (56.8%) |  |  | 34 (52.3%) | 101 (51.8%) |  |
| No | 125 (48.1%) | 71 (52.6%) | 54 (43.2%) |  |  | 31 (47.7%) | 94 (48.2%) |  |
| Primary TSR |  |  |  |  |  |  |  | **0.008** |
| ≥ 50% | 135 (51.9%) |  |  |  |  | 43 (66.1%) | 92 (47.2%) |  |
| ＜50% | 125 (48.1%) |  |  |  |  | 22 (33.9%) | 103 (52.8%) |  |

TSR tumor-stromal ratio, NTSR nodal tumor-stromal ratio

^a^ Bold indicates values with a significant difference *P <*0.05

^b^ The ypTNM staging system is according to the 8^th^ edition AJCC TNM system

^C^ Tumor location were classified according to the Japanese classification of gastric carcinoma (3rd English edition)

**Table S2** Kaplan-Meier survival analysis result of patients overall survival

| Variables | N | N of death | 3-year survival rate (%) | 5-year survival rate (%) | Long-rank test χ^2^ | *P*^a^ value |
| --- | --- | --- | --- | --- | --- | --- |
| Age |  |  |  |  | 7.681 | 0.053 |
| ＜45 | 32 (12.3%) | 11 (34.4%) | 68.4 | 63.1 |  |  |
| 45~59 | 86 (33.1%) | 45 (52.3%) | 63.8 | 44.4 |  |  |
| 60~74 | 117 (45.0%) | 62 (53.0%) | 53.8 | 43.6 |  |  |
| ≥75 | 25 (9.6%) | 17 (68.0%) | 35.6 | 29.6 |  |  |
| Gender |  |  |  |  | 1.336 | 0.248 |
| Male | 163 (62.7%) | 89 (54.6%) | 54.4 | 41.0 |  |  |
| Female | 97 (37.3%) | 46 (47.4%) | 61.7 | 50.4 |  |  |
| pN status |  |  |  |  | 39.374 | ＜0.001 |
| N1 | 83 (31.9%) | 26 (31.3%) | 72.3 | 67.4 |  |  |
| N2 | 77 (29.6%) | 34 (41.2%) | 67.2 | 53.0 |  |  |
| N3a | 68 (26.2%) | 47 (69.1%) | 45.5 | 28.3 |  |  |
| N3b | 32 (12.3%) | 28 (87.5%) | 18.8 | 11.3 |  |  |
| pT status |  |  |  |  | 7.372 | 0.007 |
| T1/T2 | 33 (12.7%) | 8 (24.2%) | 78.1 | 68.4 |  |  |
| T3/T4 | 227 (87.3%) | 127 (55.9%) | 54.1 | 41.6 |  |  |
| ypTNM staging^b^ |  |  |  |  | 9.708 | 0.008 |
| Ⅰ | 13 (5.0%) | 3 (23.1%) | 76.9 | 76.9 |  |  |
| Ⅱ | 33 (12.7%) | 9 (27.3%) | 81.4 | 64.1 |  |  |
| Ⅲ | 214 (82.3%) | 123 (57.5%) | 52.2 | 40.6 |  |  |
| Differentiation |  |  |  |  | 4.225 | 0.040 |
| Well/Moderately | 49 (18.8%) | 19 (38.8%) | 67.3 | 61.8 |  |  |
| Poor | 211 (81.2%) | 116 (55.0%) | 54.7 | 40.1 |  |  |
| Tumor location^c^ |  |  |  |  | 3.894 | 0.273 |
| Upper | 74 (28.5%) | 40 (54.0%) | 51.4 | 42.9 |  |  |
| Middle | 66 (25.4%) | 32 (48.5%) | 62.1 | 48.8 |  |  |
| Low | 113 (43.5%) | 57 (50.4%) | 59.9 | 45.8 |  |  |
| Total | 7 (2.7%) | 6 (85.7%) | 28.6 | 14.3 |  |  |
| Tumor length (cm) |  |  |  |  | 14.168 | ＜0.001 |
| ＜5 | 147 (56.5%) | 62 (42.2%) | 66.5 | 57.2 |  |  |
| ≥5 | 113 (43.5%) | 73 (64.6%) | 45.0 | 29.9 |  |  |
| CEA (ng/ml) |  |  |  |  | 1.076 | 0.300 |
| ≤5.0 | 215 (83.0%) | 108 (50.2%) | 57.1 | 46.7 |  |  |
| ＞5.0 | 44 (17.0%) | 27 (61.4%) | 56.6 | 32.6 |  |  |
| Postoperative chemotherapy |  |  |  |  | 9.771 | 0.002 |
| Yes | 135 (51.9%) | 56 (41.5%) | 65.7 | 54.6 |  |  |
| No | 125 (48.1%) | 79 (63.2%) | 47.9 | 34.9 |  |  |
| Primary TSR |  |  |  |  | 6.140 | 0.013 |
| ≥ 50% | 135 (51.9%) | 60 (44.4%) | 62.5 | 49.2 |  |  |
| ＜50% | 125 (48.1%) | 75 (60.0%) | 51.2 | 39.3 |  |  |
| NTSR |  |  |  |  | 11.873 | 0.001 |
| ＞60% | 65 | 23 (35.4%) | 75.3 | 59.0 |  |  |
| ≤60% | 195 | 112 (57.4%) | 51.1 | 39.8 |  |  |

*OS* overall survival, TSR tumor-stromal ratio, NTSR nodal tumor-stromal ratio

^a^ Bold indicates values with a significant difference *P <*0.05

^b^ The ypTNM staging system is according to the 8^th^ edition AJCC TNM system

^C^ Tumor location were classified according to the Japanese classification of gastric carcinoma (3rd English edition)

**Table S3** The Cox univariate and multivariate ananlysis for disease-free survival on primary cohort

| Variables | Univariates analysis | | |  | Multivariates analysis of PTSR | | |  | Multivariates analysis of NTSR | | |  | Multivariates analysis of PTSR and NTSR | | |
| --- | --- | --- | --- | --- | --- | --- | --- | --- | --- | --- | --- | --- | --- | --- | --- |
|  | HR | 95% CI | *P*^a^-value |  | HR | 95% CI | *P*^a^-value |  | HR | 95% CI | *P*^a^-value |  | HR | 95% CI | *P*^a^-value |
| Age |  |  | 0.832 |  |  |  |  |  |  |  |  |  |  |  |  |
| ＜45 | 0.706 | 0.215-2.314 |  |  |  |  |  |  |  |  |  |  |  |  |  |
| 45~60 | 0.854 | 0.319-2.289 |  |  |  |  |  |  |  |  |  |  |  |  |  |
| 60~75 | 1.023 | 0.395-2.649 |  |  |  |  |  |  |  |  |  |  |  |  |  |
| ＞75 | 1.000 |  |  |  |  |  |  |  |  |  |  |  |  |  |  |
| Gender |  |  | 0.235 |  |  |  |  |  |  |  |  |  |  |  |  |
| Male | 1.000 |  |  |  |  |  |  |  |  |  |  |  |  |  |  |
| Female | 0.713 | 0.409-1.245 |  |  |  |  |  |  |  |  |  |  |  |  |  |
| pN status |  |  | 0.062 |  |  |  |  |  |  |  |  |  |  |  |  |
| N1 | 0.353 | 0.154-0.810 |  |  |  |  |  |  |  |  |  |  |  |  |  |
| N2 | 0.526 | 0.240-1.156 |  |  |  |  |  |  |  |  |  |  |  |  |  |
| N3a | 0.748 | 0.344-1.623 |  |  |  |  |  |  |  |  |  |  |  |  |  |
| N3b | 1.000 |  |  |  |  |  |  |  |  |  |  |  |  |  |  |
| pT status |  |  | 0.059 |  |  |  | 0.069 |  |  |  | 0.062 |  |  |  | 0.062 |
| T1/T2 | 1.000 |  |  |  | 0.340 | 0.106-1.089 |  |  | 0.330 | 0.103-1.058 |  |  | 0.330 | 0.103-1.058 |  |
| T3/T4 | 3.062 | 0.957-9.799 |  |  | 1.000 |  |  |  | 1.000 |  |  |  | 1.000 |  |  |
| ypTNM staging^b^ |  |  | 0.307 |  |  |  |  |  |  |  |  |  |  |  |  |
| Ⅰ | 0.277 | 0.038-2.007 |  |  |  |  |  |  |  |  |  |  |  |  |  |
| Ⅱ | 0.671 | 0.288-1.566 |  |  |  |  |  |  |  |  |  |  |  |  |  |
| Ⅲ | 1.000 |  |  |  |  |  |  |  |  |  |  |  |  |  |  |
| Differentiation |  |  | 0.740 |  |  |  |  |  |  |  |  |  |  |  |  |
| Well/Moderately | 1.118 | 0.579-2.157 |  |  |  |  |  |  |  |  |  |  |  |  |  |
| Poor | 1.000 |  |  |  |  |  |  |  |  |  |  |  |  |  |  |
| Tumor location^c^ |  |  | 0.631 |  |  |  |  |  |  |  |  |  |  |  |  |
| Upper | 1.000 |  |  |  |  |  |  |  |  |  |  |  |  |  |  |
| Middle | 1.523 | 0.352-6.594 |  |  |  |  |  |  |  |  |  |  |  |  |  |
| Low | 0.791 | 0.417-1.499 |  |  |  |  |  |  |  |  |  |  |  |  |  |
| Total | 1.132 | 0.583-2.198 |  |  |  |  |  |  |  |  |  |  |  |  |  |
| Tumor length (cm) |  |  | **0.043** |  |  |  |  |  |  |  |  |  |  |  |  |
| ＜5 | 1.000 |  |  |  |  |  |  |  |  |  |  |  |  |  |  |
| ≥5 | 1.703 | 1.016-2.854 |  |  |  |  |  |  |  |  |  |  |  |  |  |
| CEA (ng/ml) |  |  | **0.020** |  |  |  | **0.028** |  |  |  | **0.047** |  |  |  | **0.047** |
| ≤5.0 | 1.000 |  |  |  | 1.000 |  |  |  | 1.000 |  |  |  | 1.000 |  |  |
| ＞5.0 | 1.979 | 1.112-3.520 |  |  | 1.906 | 1.071-3.393 |  |  | 1.795 | 1.007-3.198 |  |  | 1.795 | 1.007-3.198 |  |
| Postoperative chemotherapy |  |  | 0.191 |  |  |  |  |  |  |  |  |  |  |  |  |
| Yes | 0.708 | 0.422-1.187 |  |  |  |  |  |  |  |  |  |  |  |  |  |
| No | 1.000 |  |  |  |  |  |  |  |  |  |  |  |  |  |  |
| Primary TSR |  |  | 0.368 |  |  |  |  |  |  |  |  |  |  |  |  |
| ≥ 50% | 1.000 |  |  |  |  |  |  |  |  |  |  |  |  |  |  |
| ＜50% | 1.267 | 0.757-2.123 |  |  |  |  |  |  |  |  |  |  |  |  |  |
| NTSR |  |  | **0.011** |  |  |  |  |  |  |  | **0.013** |  |  |  | **0.013** |
| ＞60% | 1.000 |  |  |  |  |  |  |  | 1.000 |  |  |  | 1.000 |  |  |
| ≤60% | 2.623 | 1.242-5.539 |  |  |  |  |  |  | 2.592 | 1.225-5.483 |  |  | 2.592 | 1.225-5.483 |  |

*DFS* disease-free survival, TSR tumor-stromal ratio, NTSR nodal tumor-stromal ratio

^a^ Bold indicates values with a significant difference *P* <0.05

^b^ The ypTNM staging system is according to the 8^th^ edition AJCC TNM system

^C^ Tumor location were classified according to the Japanese classification of gastric carcinoma (3rd English edition)

**Table S4** Patient demographics and clinical characteristics

| Patient’s Characteristics | Total (n=389) | Primary cohort (n=260) | Validation cohort (n=129) | *P*^a^ value |
| --- | --- | --- | --- | --- |
| Age |  |  |  | 0.893 |
| ＜45 | 51 (13.1%) | 32 (12.3%) | 19 (14.7%) |  |
| 45~60 | 130 (33.4%) | 86 (33.1%) | 44 (34.1%) |  |
| 60~75 | 171 (44.0%) | 117 (45.0%) | 54 (41.9%) |  |
| ＞75 | 37 (9.5%) | 25 (9.6%) | 12 (9.3%) |  |
| Gender |  |  |  | 0.670 |
| Male | 241 (62.0%) | 163 (62.7%) | 78 (60.5%) |  |
| Female | 148 (38.0%) | 97 (37.3%) | 51 (39.5%) |  |
| pN status |  |  |  | 0.599 |
| N1 | 126 (32.4%) | 83 (31.9%) | 43 (33.3%) |  |
| N2 | 118 (30.3%) | 77 (29.6%) | 41 (31.8%) |  |
| N3a | 103 (26.5%) | 68 (26.2%) | 35 (27.1%) |  |
| N3b | 42 (10.8%) | 32 (12.3%) | 10 (7.8%) |  |
| pT status |  |  |  | 0.729 |
| T1/T2 | 51 (13.1%) | 33 (12.7%) | 18 (14.0%) |  |
| T3/T4 | 338 (86.9%) | 227 (87.3%) | 111 (86.0%) |  |
| ypTNM staging^b^ |  |  |  | 0.935 |
| Ⅰ | 19 (4.9%) | 13 (5.0%) | 6 (4.7%) |  |
| Ⅱ | 51 (13.1%) | 33 (12.7%) | 18 (14.0%) |  |
| Ⅲ | 319 (82.0%) | 214 (82.3%) | 105 (81.4%) |  |
| Differentiation |  |  |  | 0.808 |
| Well/Moderately | 72 (18.5%) | 49 (18.8%) | 23 (17.8%) |  |
| Poor | 317 (81.5%) | 211 (81.2%) | 106 (82.2%) |  |
| Tumor location^c^ |  |  |  | 0.844 |
| Upper | 114 (29.3%) | 74 (28.5%) | 40 (31.0%) |  |
| Middle | 100 (25.7%) | 66 (25.4%) | 34 (26.4%) |  |
| Low | 166 (42.7%) | 113 (43.5%) | 53 (41.1%) |  |
| Total | 9 (2.3%) | 7 (2.7%) | 2 (1.6%) |  |
| Tumor length (cm) |  |  |  | 0.877 |
| ＜5 | 221 (56.8%) | 147 (56.5%) | 74 (57.4%) |  |
| ≥5 | 168 (43.2%) | 113 (43.5%) | 55 (42.6%) |  |
| CEA (ng/ml) |  |  |  | 0.987 |
| ≤5.0 | 322 (83.0%) | 215 (83.0%) | 107 (82.9%) |  |
| ＞5.0 | 66 (17.0%) | 44 (17.0%) | 22 (17.1%) |  |
| Postoperative chemotherapy |  |  |  | 0.567 |
| Yes | 198 (50.9%) | 135 (51.9%) | 63 (48.8%) |  |
| No | 191 (49.1%) | 125 (48.1%) | 66 (51.2%) |  |
| Primary TSR |  |  |  | 0.775 |
| ≥ 50% | 200 (51.4%) | 135 (51.9%) | 65 (50.4%) |  |
| ＜50% | 189 (48.6%) | 125 (48.1%) | 64 (49.6%) |  |
| NTSR |  |  |  | 0.245 |
| ＞60% | 303 (77.9%) | 207 (79.6%) | 96 (74.4%) |  |
| ≤ 60% | 86 (22.1%) | 53 (20.4%) | 33 (25.6%) |  |

TSR tumor-stromal ratio, NTSR nodal tumor-stromal ratio

^a^ Bold indicates values with a significant difference *P <*0.05

^b^ The ypTNM staging system is according to the 8^th^ edition AJCC TNM system

^C^ Tumor location were classified according to the Japanese classification of gastric carcinoma (3rd English edition)

**Fig. S1** Detailed information about the patient selection process in both primary cohort and

validation cohort

**
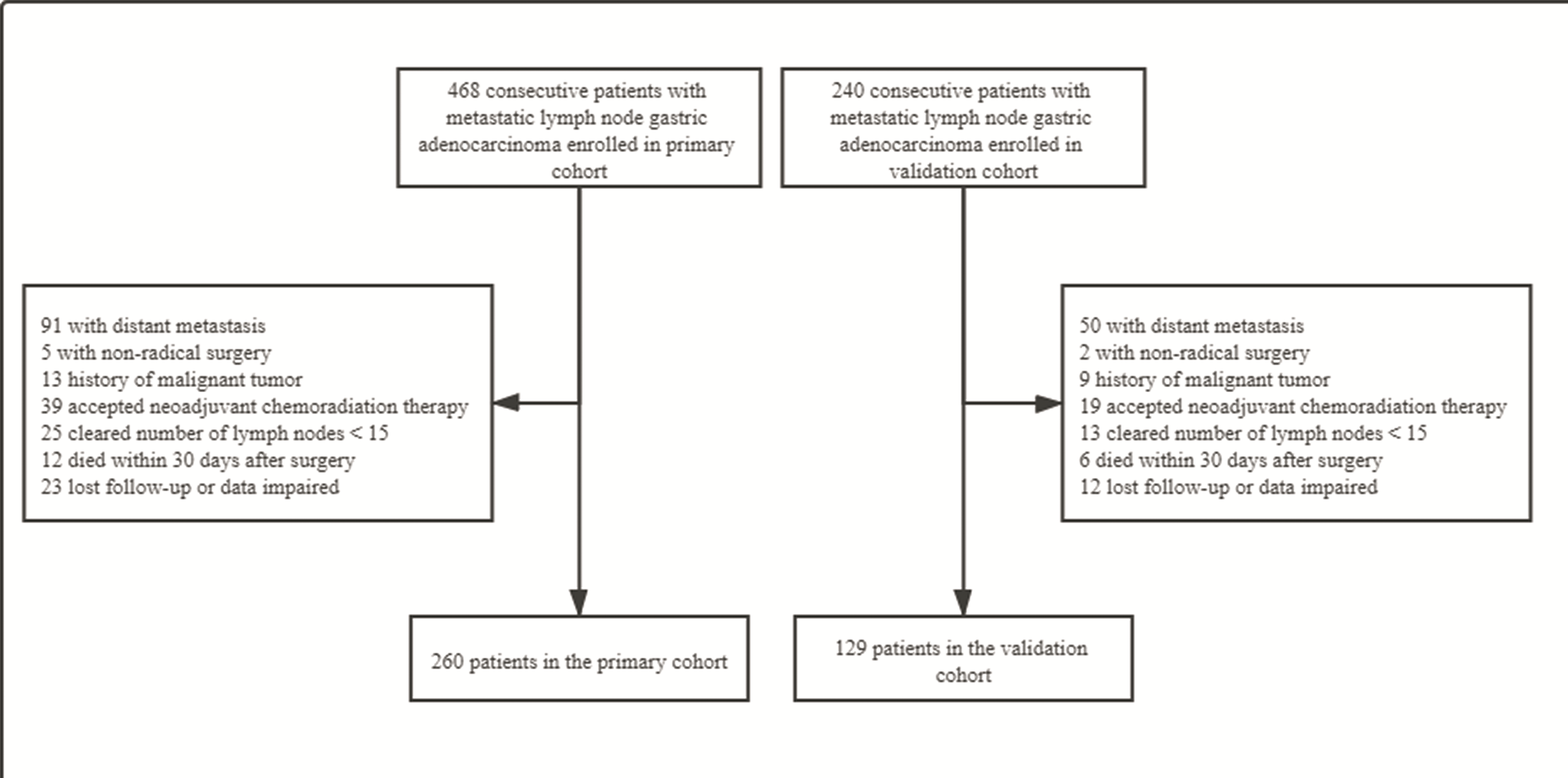
**

**Fig. S2** Pathological evaluation of TSR. Haematoxylin and eosin (H&E) stained 4 (m paraffin sections. The TSR were evaluated at the most invasive part of primary gastric cancer. Tumor cells must be present at all borders (red arrows). A Typical example of primary gastric cancer TSR ＜ 50%. B Typical example of primary gastric cancer TSR ≥ 50%. C~F Typical example of metastatic lymph nodes TSR 20%~80%.

**
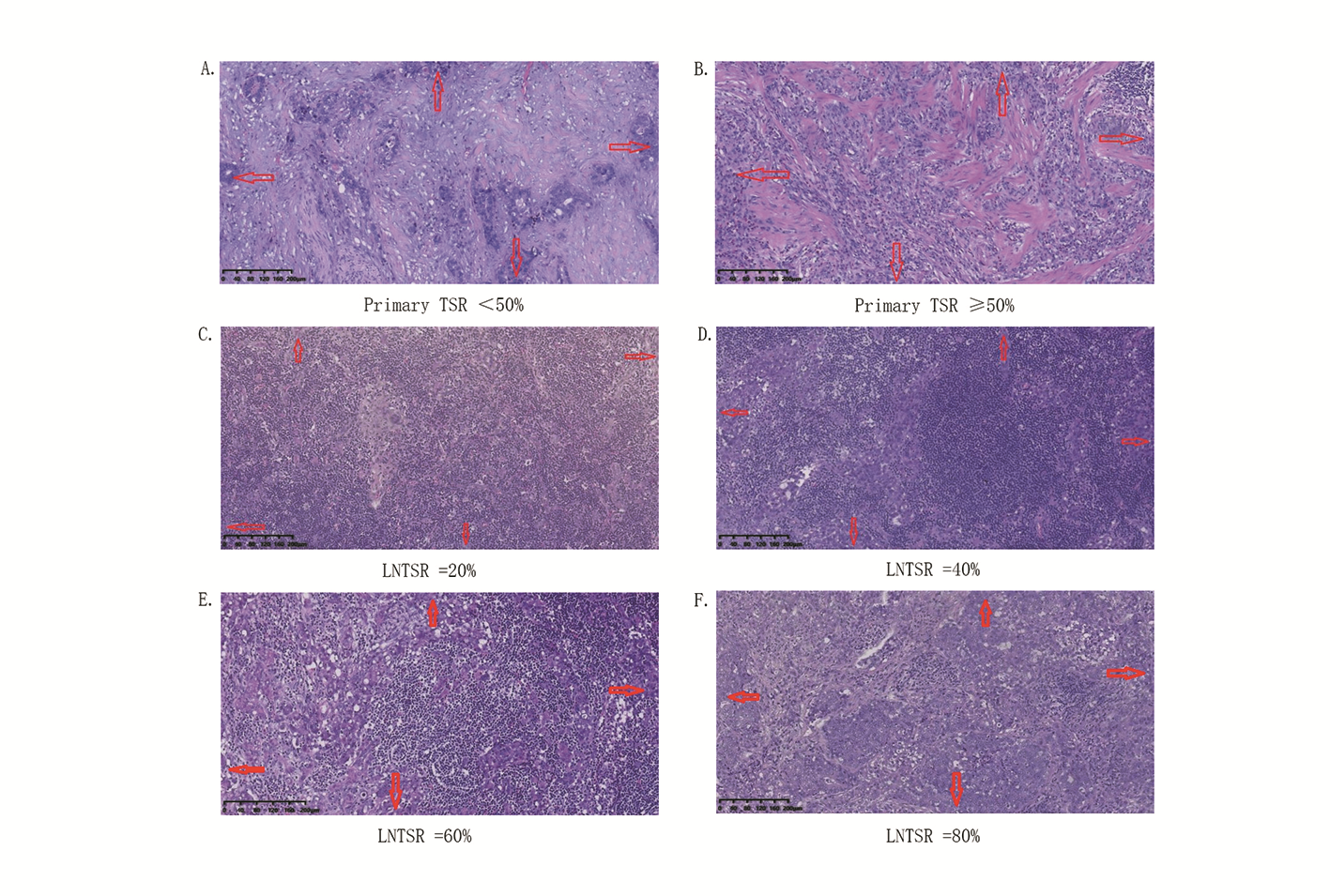
**
